# hSSB1 associates with and promotes stability of the BLM helicase

**DOI:** 10.1186/s12867-017-0090-3

**Published:** 2017-05-15

**Authors:** Laura V. Croft, Nicholas W. Ashton, Nicolas Paquet, Emma Bolderson, Kenneth J. O’Byrne, Derek J. Richard

**Affiliations:** 1 School of Biomedical Research, Institute of Health and Biomedical Innovation at the Translational Research Institute, Queensland University of Technology, 37 Kent Street, Woolloongabba, QLD 4102 Australia; 2 Princess Alexandra Hospital, 199 Ipswich Road, Woolloongabba, QLD 4102 Australia

**Keywords:** HSSB1, BLM, DNA damage, DNA repair, Double strand breaks, Replication stress

## Abstract

**Background:**

Maintenance of genome stability is critical in human cells. Mutations in or loss of genome stability pathways can lead to a number of pathologies including cancer. hSSB1 is a critical DNA repair protein functioning in the repair and signalling of stalled DNA replication forks, double strand DNA breaks and oxidised DNA lesions. The BLM helicase is central to the repair of both collapsed DNA replication forks and double strand DNA breaks by homologous recombination.

**Results:**

In this study, we demonstrate that hSSB1 and BLM helicase form a complex in cells and the interaction is altered in response to ionising radiation (IR). BLM and hSSB1 also co-localised at nuclear foci following IR-induced double strand breaks and stalled replication forks. We show that hSSB1 depleted cells contain less BLM protein and that this deficiency is due to proteasome mediated degradation of BLM. Consequently, there is a defect in recruitment of BLM to chromatin in response to ionising radiation-induced DSBs and to hydroxyurea-induced stalled and collapsed replication forks.

**Conclusions:**

Our data highlights that BLM helicase and hSSB1 function in a dynamic complex in cells and that this complex is likely required for BLM protein stability and function.

**Electronic supplementary material:**

The online version of this article (doi:10.1186/s12867-017-0090-3) contains supplementary material, which is available to authorized users.

## Background

The maintenance of genome stability is an essential requirement for cellular homeostasis. The detection and repair of DNA damages is an important aspect of this process and is facilitated by proteins that comprise a number of distinct, while over-lapping, enzymatic repair pathways [1].

Homologous recombination (HR) is a major DNA repair pathway that may be employed for the repair of double-strand DNA breaks (DSBs) during S and G2 of the cell cycle [2]. Here, 5′ DSB ends are resected to generate long stretches of single-stranded DNA (ssDNA). These ssDNA strands may subsequently invade a sister chromatid, guided by Rad51, where they are extended via complementarity [3]. Second-end capture then allows the invading strand to re-anneal with the non-invading DSB end, leaving two interlaced sister chromatids [4]. Enzymatic processing of the intervening junctions (Holliday junctions) subsequently yields two intact dsDNA strands. HR can also be used for the repair of stalled and collapsed replication forks [5, 6]. In these cases, the Rad51-nucleated ssDNA strand is generated either by resection of the ‘one-ended’ DSB formed by fork collapse [7], or from the nascent leading strand of an otherwise intact, although stalled, replication fork [6]. The nucleated strand can then re-invade the same chromatid and replication can continue without need for second-end capture [5, 6].

The remodelling of duplex DNA, as well as the migration of fork and Holliday junctions, is a central requirement of HR and is achieved by a number of DNA helicases. Bloom syndrome protein (BLM), a member of the RecQ family of helicases, is one such enzyme, with roles in HR, as well as telomere maintenance and replication [8, 9]. The enzymatic activity of BLM is achieved through its evolutionarily conserved helicase domain, a feature it shares in humans with the related helicases WRN, RECQ4, RECQ1 and RECQ5β [10]. During the initiation of HR, BLM associates with the Mre11–Rad50–Nbs1 (MRN) complex and with either the Exo1 or CtIP nucleases to promote end resection and the generation of an invading ssDNA strand [9]. In addition, BLM is required for the dissolution of double Holliday junctions, during the later stages of HR [11]. Here, BLM is able to converge both Holliday junctions into a hemi-catenane structure, which is subsequently cleaved by its binding partner TOPIIIα, generating two non-crossover products [11]. BLM is also thought to be required for the restart of stalled replication forks, where it is proposed to stimulate fork regression and Holliday junction formation [12]. This activity may result in the displacement of the nascent leading strand, generating a stretch of ssDNA that could subsequently be used for strand invasion [13].

Single-stranded DNA-binding (SSB) proteins are additional fundamental components of DNA repair pathways [14]. These can be subdivided into the “simple SSBs”, which are composed of one polypeptide, as well as the higher-order “replication protein A (RPA)”-like SSBs [15]. RPA is the most studied SSB family member in humans and is a central factor in many DNA repair processes [14–17]. Humans also encode a number of simple SSBs, including the more recently described human single-stranded DNA-binding protein 1 (hSSB1) [18]. hSSB1 has previously been found to function in HR-mediated DSB repair, where it is involved in the initial detection of lesions, as well as the stimulation of end-resection by MRN and Exo1 [19–21]. hSSB1 also has described roles in the response to replication disruption and promotes replication fork restart following polymerase inhibition [22].

In this study we demonstrate that in addition to these roles, hSSB1 can form a dynamic complex with BLM. Loss of hSSB1 results in a decreased stability of BLM polypeptide and consequently a failure in BLM accumulation at sites of DNA damage and stalled replication.

## Methods

### Cell culture and treatments

U2OS and HeLa cell lines were obtained from Sigma-Aldrich, European Collection of Authenticated Cell Cultures and were maintained at 37 °C in Roswell Park Memorial Institute (RPMI) medium supplemented with 10% fetal bovine serum. For ionising radiation treatment, cells were exposed to 6 Gy of gamma radiation generated from a cesium-source irradiator (Gammacell 40 Exactor) and were harvested at the indicated time points after treatment. Camptothecin (1 μM; Sigma-Aldrich) was added to the culture media and cells were incubated for 4 h. Hydroxyurea (HU; 2 mM; Sigma-Aldrich) was added to the culture media and cells were incubated as indicated after which cells were harvested for immunoblotting or fixed for immunofluorescence. For inhibition of the proteasome, cells were grown in RPMI supplemented with 10 μM MG132 (SelleckChem) as indicated. Cycloheximide (CHX) (2 mg/ml; Sigma-Aldrich) was added to the culture media to inhibit protein synthesis for the time indicated.

### siRNA and plasmid transfections

Stealth small interfering RNA (siRNA) (Thermo-Fisher) 5′-GCCCUUCCAGCAACCCUGUUAGUAA-3 and esiRNA (Sigma-Aldrich) were used as indicated to transiently deplete hSSB1. Transfection of siRNA was carried out using Lipofectamine RNAiMax (Thermo-Fisher) at a final concentration of 40 nM for stealth siRNA and 20 nM for esiRNA. Subsequent treatments were performed at 72 h post siRNA transfection.

WT and F98A 3× FLAG hSSB1 plasmids have been described previously [23]. HA-tagged siRNA-resistant hSSB1 was generated from a previously described siRNA resistant 3× FLAG hSSB1 construct [24] by site directed mutagenesis, which was performed using a described methodology [25] and the following primers: (F) 5-**TACCCATACGATGTTCCAGATTACGCT**GCGATCGCCATGACGACGGAGACC-3′, (R) 5′**AGCGTAATCTGGAACATCGTATGGGTA**CATGGTGGCAGATCTCCTCGGTACCGG-3′. For the rescue experiment, HA-tagged siRNA-resistant hSSB1 was transiently transfected into cells that had previously been depleted of endogenous hSSB1. Plasmids were transfected using Fugene HD (Promega) transfection reagent.

### Real time qPCR

For real time quantitative RT-PCR, RNA was isolated using the RNeasy kit (Qiagen) and 1 μg of RNA was used for cDNA synthesis using the RTPCR kit (Thermo-Fisher). qRTPCR was performed using the ViiA7 system (ABI). The primers used were as follows: BLM FWD 5′-GCAGCGATGTGATTTGCATC-3′, BLM REV 5′-ATTCAGCTTCTTCCAAATTCTTCTT-3′, hSSB1 FWD 5′-AGCCAAACCCAGAGTACAGC-3′, hSSB1 REV 5′- CTGGTTCTCAGAGGCTGGAG-3′, 7SL FWD 5′-ATCGGGTGTCCGCACTAAGTT-3′, 7SL REV 5′-CAGCACGGGAGTTTTGACCT-3′.

### Cell lysis, immunoblotting and antibodies

HeLa and U2OS whole cell lysates were prepared by resuspension of cells in radioimmunoprecipitation (RIPA) buffer, followed by sonication as described previously [24]. Samples were then electrophoresed on 4–12% Bis–Tris Plus Bolt precast gels (Thermo-Fisher) and proteins transferred onto nitrocellulose membranes prior to incubation with primary antibodies.

The hSSB1 antibody was purified from sheep anti-serum as described previously [18]. Rabbit and goat antibodies against BLM were purchased from Bethyl (cat# A310-029A) and used for immunoprecipitation and western blotting. A BLM antibody was also purchased from Sigma-Aldrich (cat# HPA005689) and used for immunofluorescence microscopy and western blotting. Commercial antibodies against RPA32 (clone 4E4, cat# 2208), SP1 (cat# 5931), pS1981 ATM (clone D6H9, cat#5883) and pol II CTD (clone 4H8, cat# 2629) were purchased from Cell Signaling Technology. MRE11 (cat# HPA002691), H3 (cat# H0164) and FLAG (clone M2, cat# F1804) antibodies were purchased from Sigma-Aldrich, the actin antibody (clone C4, cat# 612656) from BD Biosciences, the INTS3 antibody (cat# A302-050) from Bethyl, the PCNA antibody (clone PC10; cat# SANTSC-56) from VWR and the RPA70 antibody from Oncogene Research Products (cat# NA13).

Primary antibodies were detected with fluorescent (IRDye 680RD or 800CW) donkey anti-mouse, rabbit, goat or rat secondary antibodies (Li-Cor) and visualised using an Odyssey Imaging system (Li-Cor). Western blot quantification was performed with ImageJ software.

### Co-immunoprecipitation

Cells were resuspended in ice-cold immunoprecipitation buffer (20 mM HEPES pH 8, 150 mM KCl, 10 mM MgCl_2_, 0.5 mM EDTA, 0.2% NP40, 0.5 mM DTT, 5% glycerol, 1× protease inhibitor cocktail (Roche) and 1× Pierce Universal Nuclease for cell lysis (Thermo-Fisher) and lysed by sonication. Samples were then incubated with magnetic protein A/G Dynabeads (Thermo-Fisher) that had firstly been incubated with 1 μg of primary antibody per mg of cell lysate. Samples were incubated with beads for 1 h at room-temperature, beads washed five times in PBS containing 0.02% Tween 20 and bound proteins eluted in loading dye prior to analysis by immunoblotting.

### Immunofluorescence microscopy

Cells were grown in optical glass bottom 96 well plates (Cellvis). Following IR or HU treatment, cells were pre-extracted for 5 min in ice-cold extraction buffer (20 mM HEPES (pH 8), 20 mM NaCl, 5 mM MgCl_2_, 1 mM ATP and 0.5% NP40). Cells were then fixed in 4% paraformaldehyde in PBS, permeabilised in 0.2% Triton and blocked with 5% goat serum. Images were taken on a Deltavision PDV microscope and analysed using Image J (FiJi) software. Pearson correlation coefficient was measured using the Deltavision SoftWoRx software version 6.5.1 (GE Healthcare Life Sciences). One-way analysis of variance was employed to assess whether a statistically significant difference between the mean Pearson correlation coefficient value (r^2^) may exist between control and treated cells using GraphPad Prism version 6 software. Post hoc analysis was then employed to compare the mean r^2^ value of each treatment group with the mean r^2^ value of control cells.

### Sub-cellular protein fractionation

Sub-cellular protein fractionation was performed using a Pierce subcellular fractionation kit (Thermo-Fisher) as per manufacturer’s instructions.

## Results

### hSSB1 associates with BLM in cells

**Fig. 1:**
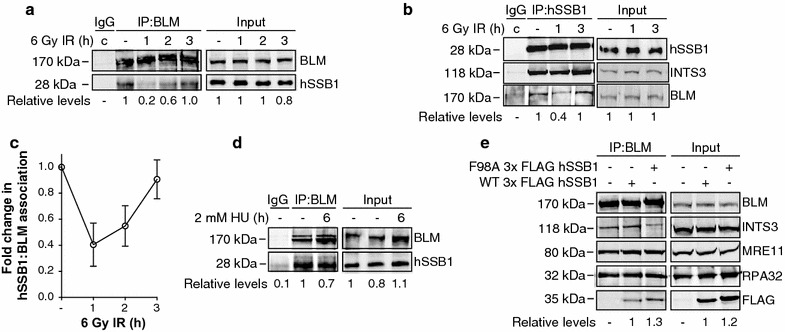
hSSB1 associates with BLM in cells. BLM (**a**) or hSSB1 (**b**)-associating proteins were immunoprecipitated from U2OS whole cell lysates prepared from cells that had been either left untreated or exposed to 6 Gy ionising radiation (IR) and harvested after the indicated time periods. Control immunoprecipitations with an isotype IgG were performed from combination (**c**) samples comprised of equal amounts of each sample. Eluted proteins and whole cell lysates were separated by electrophoresis and immunoblotted with antibodies against BLM, hSSB1 and INTS3 (**b** only). hSSB1 (**a**) or BLM (**b**) levels were determined by densitometry, normalised to the levels of BLM (**a**) or hSSB1 (**b**) as well as to the level of input protein and expressed relative to the untreated lane. **c** Line graph illustrating hSSB1: BLM association from three independent repeats of **a** and **b**. **d** BLM was immunoprecipitated from whole cell lysates, prepared from U2OS cells that were either untreated or had been treated with 2 mM hydroxyurea (HU) for 6 h. Eluted proteins and whole cell lysate samples (input) were immunoblotted with antibodies against BLM and hSSB1. hSSB1 levels were determined and expressed as per (**a**). **e** U2OS cells were transfected with plasmids encoding wild type (WT) or F98A 3× FLAG hSSB1, 24 h prior to cell lysis and immunoprecipitation of BLM-association proteins. Eluent was immunoblotted with antibodies against BLM, FLAG, INTS3, MRE11, RPA70 and RPA32. FLAG levels were determined by densitometry and normalised to the levels of BLM

Human single stranded DNA-binding protein 1 is a central cellular component required for the repair of double-strand breaks by HR, as well as the repair and restart of stalled and collapsed DNA replication forks [18, 20–22]. Recruitment kinetics of hSSB1 provide evidence for both early and late functions in these repair processes [18, 20, 21]. hSSB1 has been identified in several protein complexes including with MRN, hOGG1, INTS3, Rad51 and p53 [20, 26–28]. The BLM helicase, like hSSB1, is involved in the repair of IR-induced DSBs and the repair of stalled DNA replication forks [8, 9, 11]. BLM is also known to interact with proteins that contain OB-folds such as the trimeric RPA complex [29, 30], RMI1 and RMI2 [31, 32], thus we wondered whether BLM might also interact with the OB-fold containing protein hSSB1. We first investigated the interaction between BLM and hSSB1 at various time points post IR treatment by immunoprecipitating BLM from U2OS whole cell lysates using an anti-BLM antibody. To exclude the possibility that DNA or RNA might mediate the interaction, we treated the cell lysates with benzonase or universal nuclease before performing the co-immunoprecipitation experiments. We consistently found that hSSB1 and BLM were present in the same protein complex in cells and that the interaction decreased at 1 h post IR and returned to basal levels by 3 h post IR (Fig. 1a, c). The reciprocal immunoprecipitation with an anti-hSSB1 antibody confirmed the interaction, as well as the dynamics of the interaction with a decrease in BLM protein in the hSSB1 complex at 1 h post IR and return to basal levels at 3 h post IR (Fig. 1b, c). We also immunoblotted for INTS3, a known hSSB1 interactor [26], which confirmed the presence of INTS3 in the precipitant. Moreover, we did not observe a similar decrease in the hSSB1–INTS3 interaction in response to IR treatment, indicating that the dissociation of BLM from the hSSB1 complex is specific to BLM. These results suggest that a BLM and hSSB1-containing protein complex exists in untreated cells and that IR treatment induces an initial dissociation of BLM and hSSB1 and re-association at 2–3 h post treatment.

Both hSSB1 and BLM have described roles in the restart of stalled DNA replication forks [12, 22]. To investigate if the interaction between hSSB1 and BLM was altered after fork stalling, we treated U2OS cells with hydroxyurea (HU) for 6 h and performed immunoprecipitation reactions in whole cell lysates. As shown in Fig. 1d, we did not observe a significant change in the interaction between BLM and hSSB1, suggesting the BLM and hSSB1 interaction is not affected by the HU treatment at the time point examined. Interestingly, the BLM antibody used in the immunoprecipitation detected 2 bands (Fig. 1a, d) although only one band is associated with the hSSB1 complex (Fig. 1b; Additional file 1: Figure S1). Further studies are required to verify if these bands represent post-translationally modified BLM protein and which species of BLM is found in complex with hSSB1.

A large proportion of the cellular hSSB1 pool is present in complex with INTS3 [26]. In order to investigate whether the interaction between BLM and hSSB1 is mediated by INTS3, 3× FLAG-tagged hSSB1 wild type (hSSB1–FLAG–WT) and INTS3-binding mutant F98A (hSSB1–FLAG–F98A) (decreases hSSB1 binding to INTS3 complex; Additional file 1: Figure S2 [23, 33]) plasmids were overexpressed in U2OS cells and protein complexes were immunoprecipitated using an anti-BLM antibody. As shown in Fig. 1e, both WT and F98A mutant hSSB1 were co-immunoprecipitated by the BLM antibody to similar levels, however the INTS3 protein did not immunoprecipitate to the same degree in the cells expressing the hSSB1–F98A mutant, suggesting that INTS3 is not required for the hSSB1–BLM interaction. It does however demonstrate for the first time that INTS3 is in complex with BLM and hSSB1 within cells. The known BLM binding partners MRE11, RPA70 and RPA32 were also detected in the BLM complex (Fig. 1e).

We further investigated whether hSSB1 and BLM may interact directly using recombinant hSSB1 (*E. coli* expressed) and BLM (Insect cell expressed) proteins. However, we were unable to detect a direct interaction between the two proteins (data not shown). Taken together, these results suggest that BLM and hSSB1 may associate in cells through an indirect means, or may require specific post-translational modifications not present in the recombinant proteins. Indeed, both hSSB1 and BLM are known to exist in phosphorylated, ubiquitinated and sumoylated (BLM only) forms [8].

### hSSB1-positive chromatin structures partially overlap with BLM-positive chromatin structures following IR- and HU- induced DNA damage

**Fig. 2:**
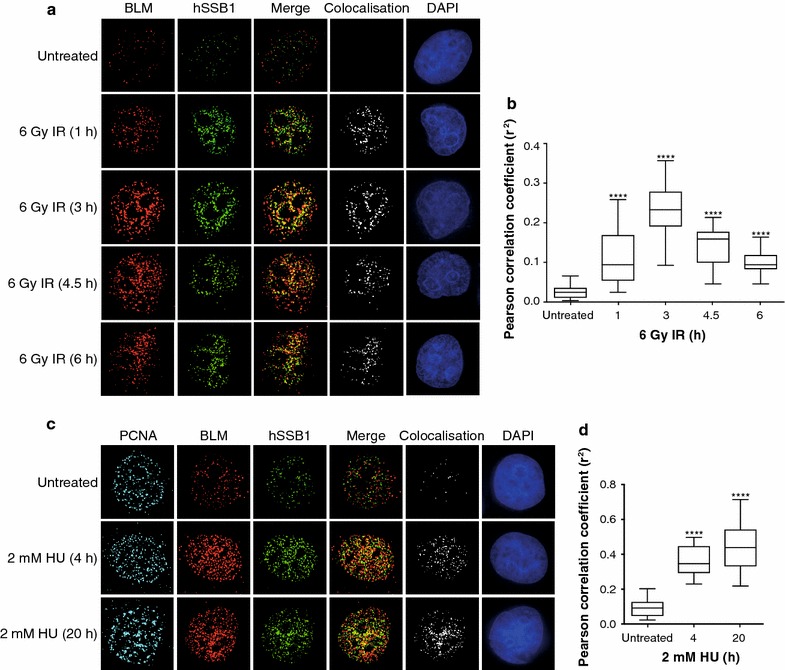
hSSB1 co-localises with BLM at IR- and HU-induced nuclear foci. **a**, **c** HeLa cells were pre-extracted prior to fixation, 0, 1, 3, 4.5 and 6 h post ionising radiation (IR) treatment (**a**) or 0, 4 and 20 h post treatment with hydroxyurea (HU) as indicated (**c**). Fixed cells were incubated with primary antibodies against BLM, hSSB1 and PCNA (**c** only), which were detected with fluorescent secondary antibodies and visualised using a DeltaVision PDV microscope. Co-localisation of BLM and hSSB1 is demonstrated by the merged image (merge) and by the analysis of images using the Image J colocalisation plugin showing only the colocalised pixels (colocalisation). DAPI was used to stain the nuclei. **b**, **d** *Box* and *Whisker plots* representing Pearson correlation coefficient (r^2^) from 24 (for **a**) and 15 (for **c**) individual cells for each time-point from **a** and **c**. One-way Anova followed by post hoc analysis was used to assess the statistical significance between the mean r^2^ values of each treatment group and untreated cells. ****p < 0.0001

Human single stranded DNA-binding protein1 and BLM have been shown to re-locate to chromatin in cells treated with DNA damaging agents and DNA replication inhibitors [18, 22, 34, 35]. To determine whether BLM and hSSB1 relocate to the same chromatin structures in response to DSB induction, we performed a time course following IR treatment in asynchronous HeLa cells. Prior to fixation at 0, 1, 3, and 6 h post IR treatment (6 Gy) cells were pre-extracted to remove the soluble nucleoplasm, leaving only the insoluble chromatin bound proteins to be immunostained and visualised. As expected, both BLM and hSSB1 showed a large increase in chromatin localisation following IR treatment, with a partial overlap between hSSB1- and BLM- positive fluorescence staining (Fig. 2a). Measurement of the Pearson correlation coefficient (r^2^) determined that the overlap between BLM- and hSSB1-positive staining significantly increased at all time points tested, with a peak at 3 h post IR (Fig. 2b).

We also investigated the localisation of BLM and hSSB1 following replication stress. HeLa cells were treated with HU for 4 h to induce mostly stalled replication forks and for 20 h to induce stalled and collapsed replication forks [6]. Prior to fixation, cells were extracted to remove the soluble nucleoplasm. To identify S-phase cells, PCNA was used as a marker. As expected, we observed an increase in BLM and hSSB1 chromatin localisation post both 4 and 20 h of HU treatment (Fig. 2c), with a significant increase in overlap between hSSB1- and BLM-positive staining at both time points (Fig. 2d).

The partial overlap between hSSB1 and BLM at IR- and HU- induced chromatin structures may represent different sub-compartments within one repair centre or a snapshot of the temporally dynamic composition of these chromatin structures [36], which is consistent with their roles in the unwinding of the DNA (BLM) and coating of the single stranded DNA (hSSB1) during the repair process. Taken together, these results suggest that although BLM and hSSB1 are part of the same DNA repair complex they may function at different sites or stages in the DSB repair process and in the restart and repair of stalled and collapsed replication forks.

### hSSB1 is required for recruitment of BLM to chromatin in response to DNA damage

**Fig. 3:**
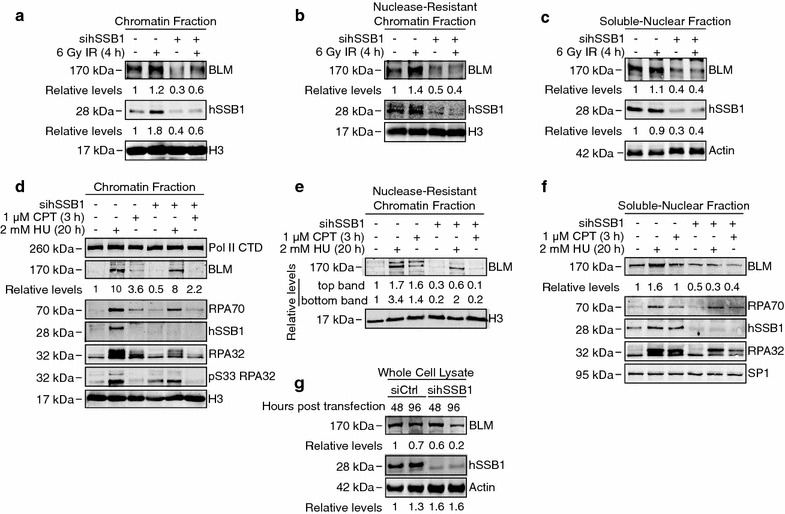
hSSB1 is required for recruitment of BLM to chromatin in response to DNA damage. **a**, **b**, **c** U2OS cells were transfected with control or hSSB1 mRNA-targeting siRNA. 72 h later, cells were then either left untreated or exposed to 6 Gy ionising radiation (IR), 4 h prior to harvesting and subcellular fractionation. Proteins from the indicated fractions were separated by electrophoresis and immunoblotted with antibodies against BLM, hSSB1, H3 (for chromatin fractions) and actin (for the soluble nuclear fraction). BLM levels were determined by densitometry, normalized to the levels of H3 or actin and expressed relative to the untreated lane. **d**, **e**, **f** U2OS cells were depleted of hSSB1 as per above and treated with 1 μM camptothecin (CPT) for 3 h or 2 mM hydroxyurea (HU) for 20, 72 h post siRNA transfection. Cells were then harvested, subcellular fractionation performed and the indicated fractions immunoblotted with antibodies against BLM, hSSB1, Pol II CTD, RPA70, pS33 RPA32, RPA32, SP1 and H3 as indicated. BLM levels were determined as per above. **g** HeLa cells were transfected with hSSB1 transcript-targetting siRNA and harvested after 48 or 96 h. Whole cell lysates were prepared and immunoblotted with antibodies against BLM, hSSB1 and actin

We and others have shown that in response to DSB formation, hSSB1 is required for both activation of signalling and proper recruitment of repair proteins at these sites [18, 19, 21, 37]. We therefore investigated whether hSSB1 depletion may affect BLM recruitment to chromatin in response to DNA damaging agents and replication stress using subcellular fractionation to separate the nuclear components. U2OS cells transfected with control or hSSB1-targeting esiRNA (Sigma-Aldrich) were irradiated to cause DSBs and harvested 4 h post treatment. As expected, BLM was found to load onto chromatin following IR treatment in control siRNA transfected cells (Fig. 3a). In contrast, in hSSB1-depleted cells, BLM chromatin loading was consistently reduced at 4 h and 3 h post IR treatment (Fig. 3a; Additional file 1: Figure S3). Moreover, we also observed consistently reduced levels of BLM chromatin loading in the nuclease-resistant chromatin fraction (Fig. 3b) and reduced protein levels in the soluble nuclear fraction (Fig. 3c), suggesting that hSSB1 may regulate BLM protein levels in the soluble nuclear fraction, consequently also affecting its recruitment to chromatin.

We have recently shown that hSSB1 is also required for recruitment of DNA repair proteins to collapsed replication forks [22]. Consistent with its roles in HR and DNA replication, BLM expression is highest during S/G2 cell cycle phases [38, 39], we hence investigated whether hSSB1 depletion may also affect BLM protein levels and recruitment to chromatin in response to replication stress, in the different nuclear compartments. HeLa cells transfected with control or hSSB1 targeting stealth siRNA (Thermo-Fisher) were treated with HU (2 mM) for 20 h to induce replication fork collapse or with camptothecin (CPT; 1 μM) for 3 h to cause replication-induced DSBs. As expected, BLM was found to load onto chromatin (tenfold and 3.6 fold increase, respectively) following HU and CPT treatment in control siRNA transfected cells (Fig. 3d). In contrast, in hSSB1-depleted cells, BLM chromatin loading was slightly, but reproducibly reduced following HU and CPT treatment (eightfold and 2.2 fold increase, respectively). Following HU treatment, RPA is phosphorylated on Ser 33 and this facilitates the response of replication forks to replication stress [40]. We also observed that loading of RPA70, RPA32 and phosphorylation of RPA32 at serine 33 (S33) were reduced in hSSB1 depleted cells (Fig. 3d). In the nuclease resistant chromatin fraction, two BLM bands were detected and although both bands showed decreased chromatin loading in hSSB1 depleted cells, a more pronounced defect was observed for the top band (Fig. 3e). Moreover, soluble nuclear levels of BLM were decreased in hSSB1-depleted cells to half the amount of control cells, and a lack of BLM protein stabilisation in response to HU treatment was also evident in hSSB1-depleted cells (Fig. 3f). Similarly, RPA 70 and RPA 32 protein levels were also decreased following hSSB1 depletion (Fig. 3f). The defect in BLM chromatin recruitment following HU treatment in absence of hSSB1 was detected in both HeLa and U2OS cells (Additional file 1: Figure S4). Consistent with this BLM defect in the nuclear fractions, total BLM protein levels were reduce by ~50% in hSSB1 depleted HeLa cells compared to control cells (Fig. 3g; Additional file 1: Figure S5). Collectively, these results suggest that hSSB1 may regulate the total cellular pool of BLM, affecting its recruitment to chromatin in response to IR-induced DSBs, replication fork collapse and replication-induced DSBs.

### hSSB1 protects BLM from proteasomal degradation

**Fig. 4:**
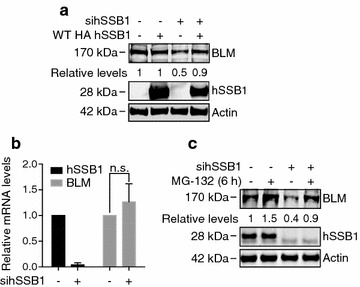
hSSB1 protects BLM from proteasomal degradation. **a** U2OS cells were transfected with control or hSSB1-depleting siRNA, 48 h prior to transfection with an siRNA-resistant HA-tagged hSSB1 plasmid (WT HA hSSB1). Cells were harvested after a further 48 h and whole cell lysates immunoblotted with antibodies against BLM, hSSB1 and actin. BLM levels were determined by densitometry, normalised to the levels of actin and expressed relative to the untreated lane. **b** U2OS cells were transfected with control or hSSB1-depleting siRNA for 72 h. BLM and hSSB1 mRNA levels were determined by real time PCR and normalised to the mRNA levels of 7SL. The graph represents the results from three independent experiments. A *t* test was used to assess statistical significance where p < 0.05 was considered significant. *n.s.* non-significant. **c** U2OS cells were transfected with control or hSSB1-depleting siRNA, 72 h prior to incubation with 10 μM MG-132 for 6 h. Cells were then lysed and whole cell lysates immunoblotted with antibodies against BLM, hSSB1 and actin. BLM levels were determined by densitometry, normalised to the levels of actin and expressed relative to the untreated lane

To further confirm our data, we performed rescue experiments using a siRNA-resistant, HA-tagged hSSB1 construct (HA-hSSB1). 48 h post hSSB1-targeting siRNA transfection, U2OS cells were transfected with the HA-hSSB1 plasmid and harvested 48 h later. Importantly, ectopic expression of hSSB1 was able to rescue BLM protein levels to near-basal levels (Fig. 4a; Additional file 1: Figure S6), indicating that BLM depletion was not due to siRNA off-target effects. Based on these findings we reasoned that hSSB1 could regulate BLM helicase by at least two mechanisms: by regulating BLM transcript, (indeed hSSB1 has recently been shown to be involved in regulation of transcription termination [41]), or by regulating BLM protein stability.

To address whether hSSB1 may affect BLM transcript levels, we performed real time quantitative PCR in U2OS cells depleted of hSSB1 using stealth and esiRNA. As shown in Fig. 4b, the average of three independent experiments, a slight, although not significant upregulation of BLM transcripts levels were observed in hSSB1-depleted cells compared to control cells, suggesting that the lack of BLM protein was not due to reduced levels of BLM transcript. The loss of the BLM polypeptide in hSSB1-depleted cells may therefore reflect a loss of protein stability. To investigate whether hSSB1 regulates BLM protein stability by preventing its proteasome-mediated degradation, we next treated U2OS cells depleted of hSSB1 with the proteasome inhibitor MG132. Indeed, MG132 treatment restored BLM protein levels in hSSB1-depleted U2OS cells (Fig. 4c; Additional file 1: Figure S6), suggesting that hSSB1 interaction with the BLM complex may protect BLM from degradation in cells.

## Discussion

BLM is an essential helicase with demonstrated roles in HR-mediated DNA repair processes [8, 9, 11]. In this study, we have identified a novel association between BLM and hSSB1, a single-stranded DNA-binding protein also known to promote HR during double-strand DNA break repair and stalled replication fork stability and repair [14, 18, 22]. Reciprocal co-immunoprecipitation experiments suggested that while BLM and hSSB1 associate in undamaged cells, the induction of double-strand DNA breaks by ionising radiation exposure results in an initial dissociation of the hSSB1–BLM complex, prior to re-association 2–3 h post treatment. These data may suggest that while hSSB1 and BLM form a complex in undamaged cells, they may function independently in the early stages of HR-mediated DSB repair. This may include during end-resection, a process that is facilitated by the BLM–DNA2–RPA–MRN and EXO1–BLM–RPA–MRN complexes [42]. Indeed, whilst we and others have suggested a role for hSSB1 in the initial stimulation of this process [18, 19], our data may suggest that hSSB1 might not remain coupled to the BLM-associated end-resection machinery subsequent to initial loading. The dissociation could also be mediated by post-translational modifications on both proteins, which could result in changes in complex formation.

The observation that hSSB1 and BLM re-associate after 2–3 h of DSB formation may however suggest a functional role in HR subsequent to end resection. Although a role for hSSB1 in later stage HR remains unclear, it should be noted that an association with the Rad51 recombinase, a known interactor of BLM, has been previously demonstrated [18]. Indeed, our observation of increased chromatin co-localisation between hSSB1 and BLM 3 and 6 h post-IR exposure support a functional relevance of their interaction in HR. It will therefore be interesting to establish whether hSSB1 and BLM function together in the regulation of Rad51-mediated strand invasion, as well as in the well-established roles of BLM in double Holliday junction dissolution [43].

A similar interaction and co-localisation of hSSB1 and BLM was also observed following replication fork disruption by HU treatment. Although the functional significance of this observation also remains unclear, roles for both of these proteins in such a response has been previously suggested [14, 22, 44–46]. This includes the BLM-mediated regression of replication forks and the generation of a ssDNA invading strand for HR-mediated fork restart by HR [12]. As hSSB1 also has been found to promote replication fork restart, the investigation of a potential role together in this process may also be of future value. The partial co-localisation between BLM and hSSB1 at nuclear structures following IR and HU treatment may be due to the fact that hSSB1 is involved at multiple stages of HR and thus likely to be present in chromatin structures that have not yet recruited BLM. Thus the duration of time in which hSSB1 and BLM are present together at a DSB site may be very short and certainly requires further investigation in the future.

The exact nature of the BLM: hSSB1 complex also remains uncertain. As we were unable to detect a direct physical interaction between recombinant BLM and hSSB1, it remains probable that other proteins instead mediate this association. This may include components of the core BLM complex (BLM–TopoIIIα–RPA–RMI1–RMI2), as well as members of the larger BASC super complex, which also includes MSH2, MSH6, MLH1, ATM, RFC and the MRN complex [35, 47]. It is indeed worth noting that a direct interaction between hSSB1 and the MRN component MRE11 has been previously established, which may represent a plausible intermediate [20]. Alternatively, we cannot exclude that a direct interaction between hSSB1 and BLM may occur in human cells, although requires specific post-translational modifications of BLM or hSSB1 that are not present in the recombinant protein. A full dissection of the BLM–hSSB1 association will however require additional experimentation.

An important observation of this study was the apparent dependence of BLM protein stability on hSSB1. Although we cannot conclude on the processes through which this occurs, it is noted that BLM levels in hSSB1-depleted cells were largely restored by proteasome inhibition with the compound MG-132. It is therefore tempting to consider that hSSB1 may promote BLM stabilisation via its interaction with one of the BLM complexes. Alternatively, hSSB1 may regulate cell signaling pathways that affect BLM post translational modifications to prevent its degradation. Indeed, BLM was recently shown to be ubiquitinated by the E3 ligase MIB1, and interaction between BLM and TopBP1 prevented MIB1–dependent degradation of BLM [39]. The interaction between hSSB1 and BLM may act in a similar manner to prevent BLM from proteasome mediated degradation. Interestingly, hSSB1 was recently shown to interact with both p53 and p21 and protect both proteins from ubiquitin-mediated degradation [28, 48]. Taken together, our current findings provide evidence for an additional, indirect means through which hSSB1, via regulation of BLM helicase, promotes DSB repair and replication fork processing.

## Conclusions

The findings described in this work suggest hSSB1 as a novel component of BLM complexes. These data thereby provide new insight into the regulation of BLM and implicate an additional means through which hSSB1 may promote genome stability in the cell.

## Additional file

**Additional file 1.** Additional figures.

## Abbreviations

ATM: ataxia telangiectasia mutated
BASC: BRCA1-associated genome surveillance complex
BLM: bloom syndrome protein
CHX: cycloheximide
CPT: camptothecin
CtIP: C-terminal binding protein interacting protein
DSB: double-strand DNA break
Exo1: exonuclease 1
Gy: gray (unit)
HR: homologous recombination
hSSB1: human single stranded DNA-binding protein 1
HU: hydroxyurea
INTS3: integrator complex subunit 3
IR: ionising radiation
MRN: Mre11–Rad50–NBS1
PCNA: proliferating cell nuclear antigen
qRT-PCR: quantitative reverse transcription polymerase chain reaction
RPA: replication protein A
RPA32: RPA 32 kDa subunit
RPA70: RPA 70 kDa subunit
SSB: single-stranded DNA-binding
TopBP1: DNA topoisomerase 2-binding protein 1
TOPO IIIα: DNA topoisomerase III alpha
WRN: Werner syndrome ATP-dependent helicase
