# Supplementary material for: hSSB1 associates with and promotes stability of the BLM helicase
Source: BMC Mol Biol. 2017 May 15;18:13. doi: 10.1186/s12867-017-0090-3 (PMC5433028; doi:10.1186/s12867-017-0090-3)
Supplement: Supplementary file 1 — Additional file 1. Additional figures. [file 12867_2017_90_MOESM1_ESM.docx]

**Supporting Information**

**hSSB1 associates with and promotes stability of the BLM helicase**

Laura V. Croft^1^, Nicholas W. Ashton^1^, Nicolas Paquet^1^, Emma Bolderson^1^, Kenneth J. O’Byrne^1,2^* and Derek J. Richard^1^*

^1^School of Biomedical Research, Institute of Health and Biomedical Innovation at the Translational Research Institute, Queensland University of Technology, 37 Kent Street, Woolloongabba, QLD 4102, Australia.

^2^Princess Alexandra Hospital, 199 Ipswich Road, Woolloongabba, QLD 4102, Australia

* To whom correspondence should be addressed: derek.richard@qut.edu.au, k.obyrne@qut.edu.au.

**CONTENT:**

Supplementary Figure 1

Supplementary Figure 2

Supplementary Figure 3

Supplementary Figure 4

Supplementary Figure 5

Supplementary Figure 6

**Figure S1: A single band representing BLM protein was identified on immunoblots following hSSB1 immunoprecipitation. (A)** HeLa whole cell lysates were prepared and incubated with protein G dynabeads bound to either hSSB1 or BLM antibodies, or an IgG isotype control, for 1 hour. Beads were then washed and bound proteins eluted and analysed by immunoblotting with antibodies against hSSB1 and BLM. Whole cell lysates were also immunoblotted with hSSB1 and BLM antibodies.

**Figure S2: hSSB1 F98A mutation disrupts binding to INTS3. (A)** HeLa cells were transiently transfected with WT or F98A 3x FLAG hSSB1, or an empty vector and harvested after 24 hours. Whole cell lysates were prepared and incubated with magnetic M2 anti-FLAG beads for 1 hour. Beads were then washed and bound proteins eluted and analysed by immunoblotting with antibodies against INTS3 and FLAG. Whole cell lysates were also immunoblotted with INTS3 and FLAG antibodies.

**Figure S3: hSSB1 depletion limits IR-induced BLM chromatin recruitment. (A and B)** HeLa cells were transfected with hSSB1-targetting Stealth siRNA or a scramble control. After 72 h, cells were exposed to 6 Gy of ionising radiation (IR), 3 hours prior to harvesting and subcellular fractionation. The nuclease-digested (Chromatin Fraction) and nuclease-resistant chromatin (Nuclease-Resistant Chromatin) fractions were analysed by immunoblotting with antibodies against hSSB1, BLM, pS1981 ATM and H3 or gamma (γ) tubulin (loading control).

**Figure S4: hSSB1 depletion prevents efficient BLM chromatin recruitment in HeLa and U2OS cells. (A and B)** U2OS (A) and HeLa (B) cells were transfected with hSSB1-targetting esiRNA or a scramble control. After 72 h, cells were further treated +/- 2 mM hydroxyurea (HU) for 20 h, prior to harvesting and subcellular fractionation. The chromatin fractions were analysed by immunoblotting with antibodies against hSSB1, as well as with antibodies against BLM, MRE11 and H3.

**Figure S5: hSSB1 depletion reduces total BLM protein levels. (A)** HeLa cells that had been depleted of hSSB1 were treated +/- 2 mM hydroxyurea (HU) for 20 h prior to harvesting. Whole cell lysates were then prepared and immunoblotted with antibodies against BLM, MRE11 and hSSB1.

**Figure S6: hSSB1 protects BLM from proteasomal degradation. (A and B)** U2OS cells were transfected with control or hSSB1-depleting siRNA, 48 hours prior to transfection with an siRNA-resistant HA (A) or 3x FLAG (B)-tagged hSSB1 plasmid. After 24 hours, cells were treated with 2 mM hydroxyurea (HU) for 4 hours and then harvested. Whole cell lysates were then prepared and immunoblotted with antibodies against BLM, hSSB1, FLAG (for B) and actin. **(C)** U2OS cells were transfected with control or hSSB1-depleting siRNA, 72 h prior to incubation with 10 μM MG-132 for 6 hours. Cells were then lysed and whole cell lysates immunoblotted with antibodies against BLM (rabbit (R) or goat (G)-generated Bethyl antibodies), hSSB1 and Actin.
